# Supplementary figures and images for: Injection site vaccinology of a recombinant vaccinia-based vector reveals diverse innate immune signatures
Source: PLoS Pathog. 2021 Jan 13;17(1):e1009215. doi: 10.1371/journal.ppat.1009215 (PMC7837487; doi:10.1371/journal.ppat.1009215)

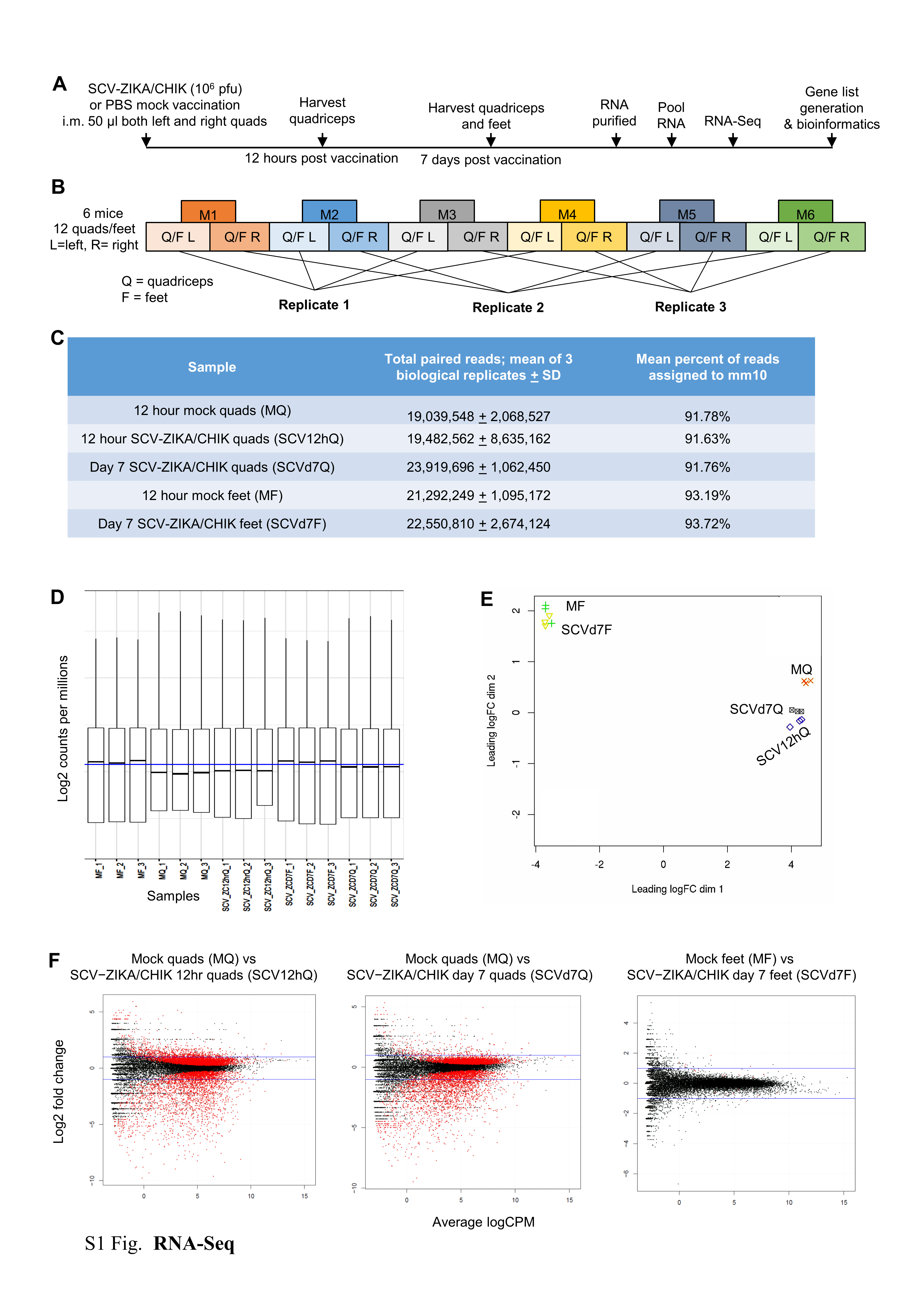

Supplement: S1 Fig — (A) Time line of experiment. (B) Pooling strategy for replicates. (C) Reads and percent of reads assigned to the mouse genome. (D) Boxplot of Log counts (normalized). Boxplots shows similar distributions of read counts amongst samples within and between groups. Boxes are 1st & 3rd quartile; whiskers range (no outliers). (E) MDS plot showing (i) clear separation between feet and quadriceps muscle groups, (ii) tight clustering of triplicates for MQ, SCVd7Q and SCV12hQ groups, (iii) clear separation between MQ, SCVd7Q and SCV12hQ groups, and (iv) poor separation between MF and SCVd7F (consistent with the low number of DEGs). (F) Smear plots of the differentially expressed genes for the three comparisons. Red–FDR <0.05. Blue lines represent fold change of 2. (TIF) [file ppat.1009215.s001.TIF]

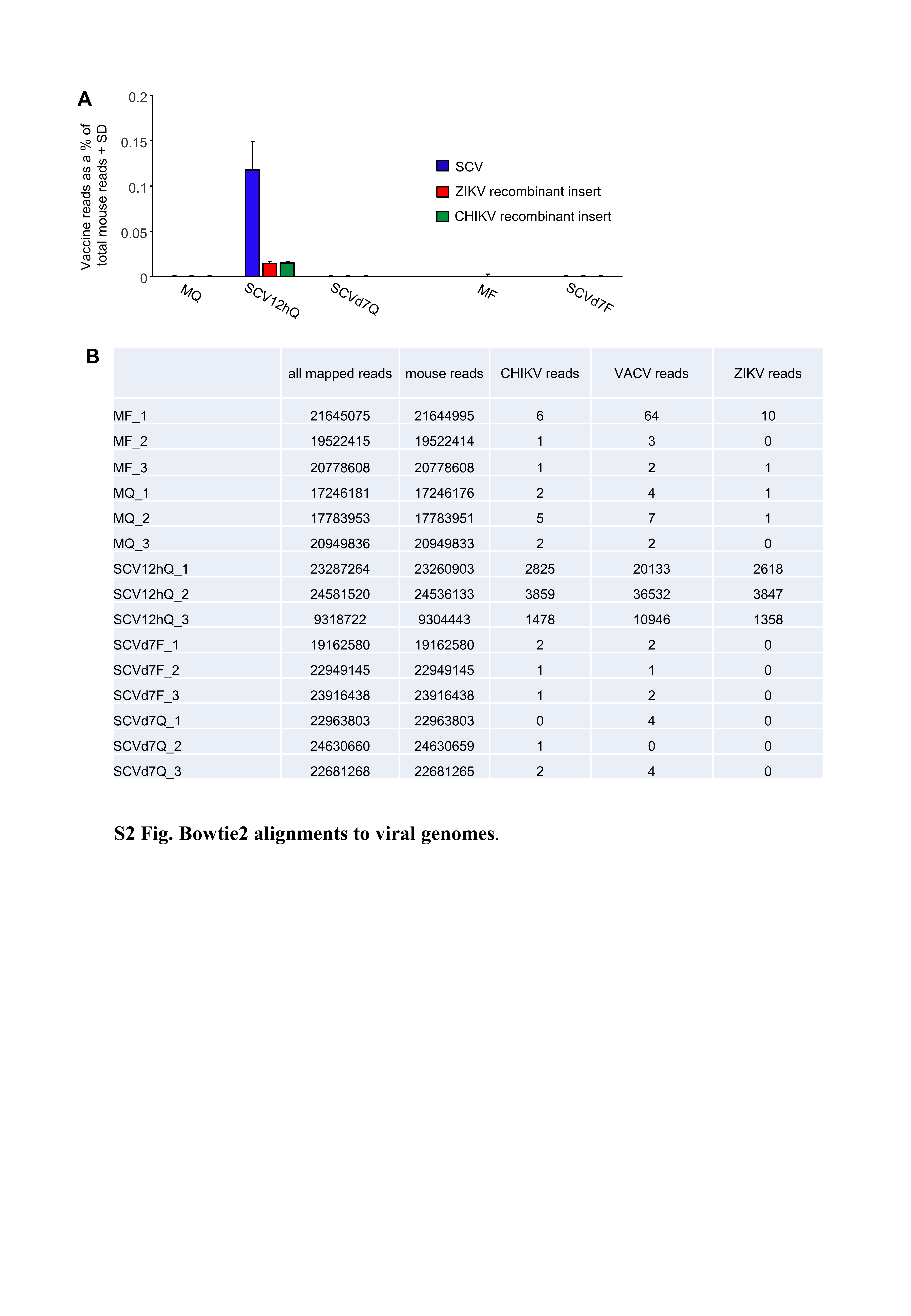

Supplement: S2 Fig — Raw FASTQ files were assessed for quality using FastQC and MultiQC tools. Sequencing adapters were trimmed using Trimmomatic (0.36.6) 1 where reads with an average quality score over a 4 base sliding window of less than 20 were removed. Trimmed reads were aligned using Bowtie2 (v2.3.4.1)2 to a combined reference that included the GRCm38 primary assembly and the GENCODE M23 gene model, Vaccinia virus Copenhagen (M35027.1), Zika virus strain Zika SPH2015 (KU321639.1), and chikungunya virus (AM258992.1). Primary proper pair reads aligned to viral features, including CDS and mature peptide features, were counted using SAMtools (v1.9). (A) Bar graph of vaccine read counts expressed as a percentage of reads aligning to the mouse genome. (B). Raw data for A. 1Bolger AM, Lohse M, Usadel B. Trimmomatic: a flexible trimmer for Illumina sequence data. Bioinformatics. 2014;30(15):2114–20. 2Langmead B, Salzberg SL. Fast gapped-read alignment with Bowtie 2. Nature Methods. 2012;9(4):357–9. (TIF) [file ppat.1009215.s002.TIF]

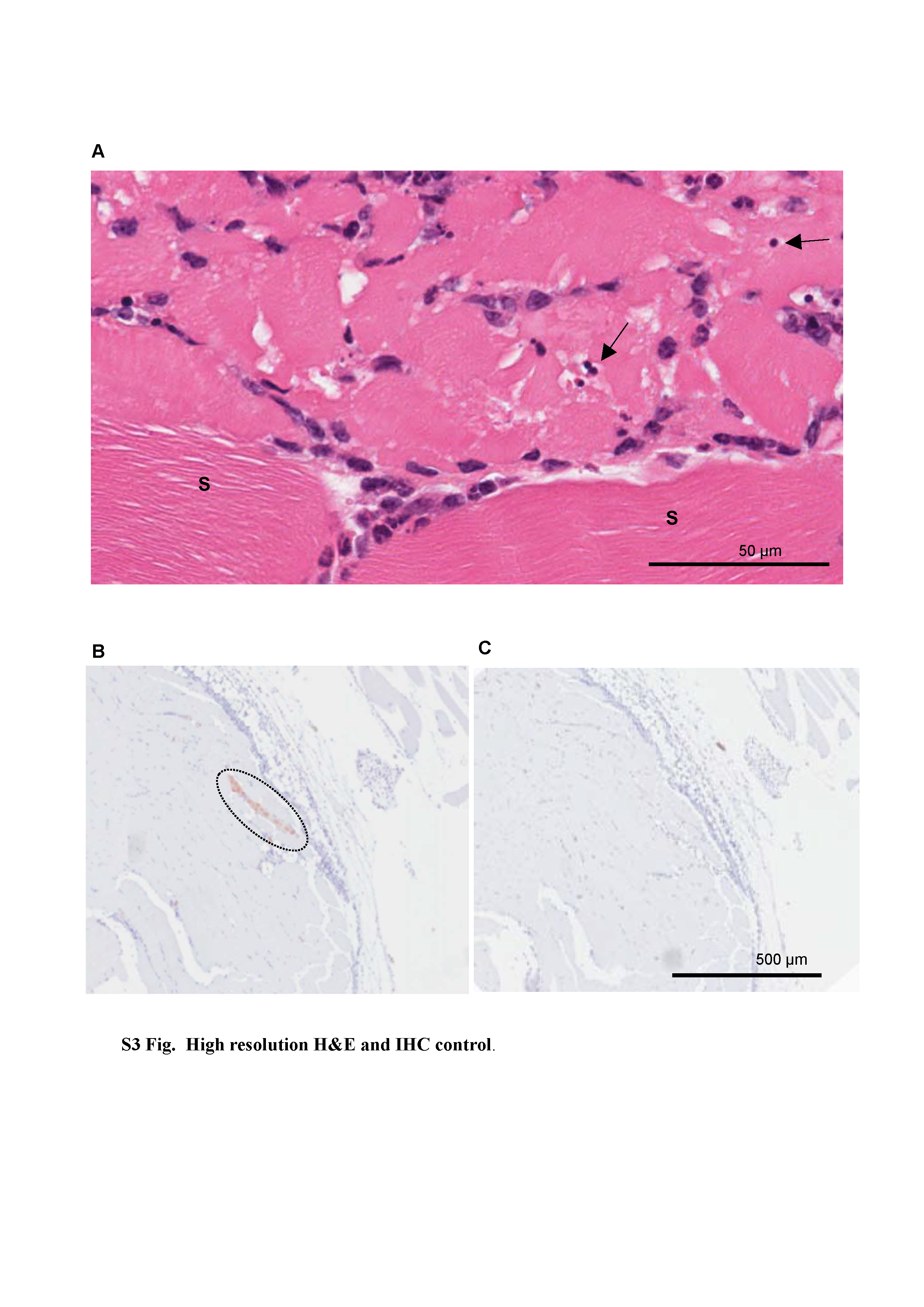

Supplement: S3 Fig — (A) High resolution image of Fig 1D showing the striations in health muscle cells (S) and above these, paler muscle cells that have lost their striated appearance. Small condensed pyknotic nuclei are indicated by arrows. (B) Expanded view of IHC staining shown in Fig 1F, with positive staining indicated by dotted oval. (C) Staining of a parallel section to that shown in B stained with a control antibody. (TIFF) [file ppat.1009215.s003.tiff]

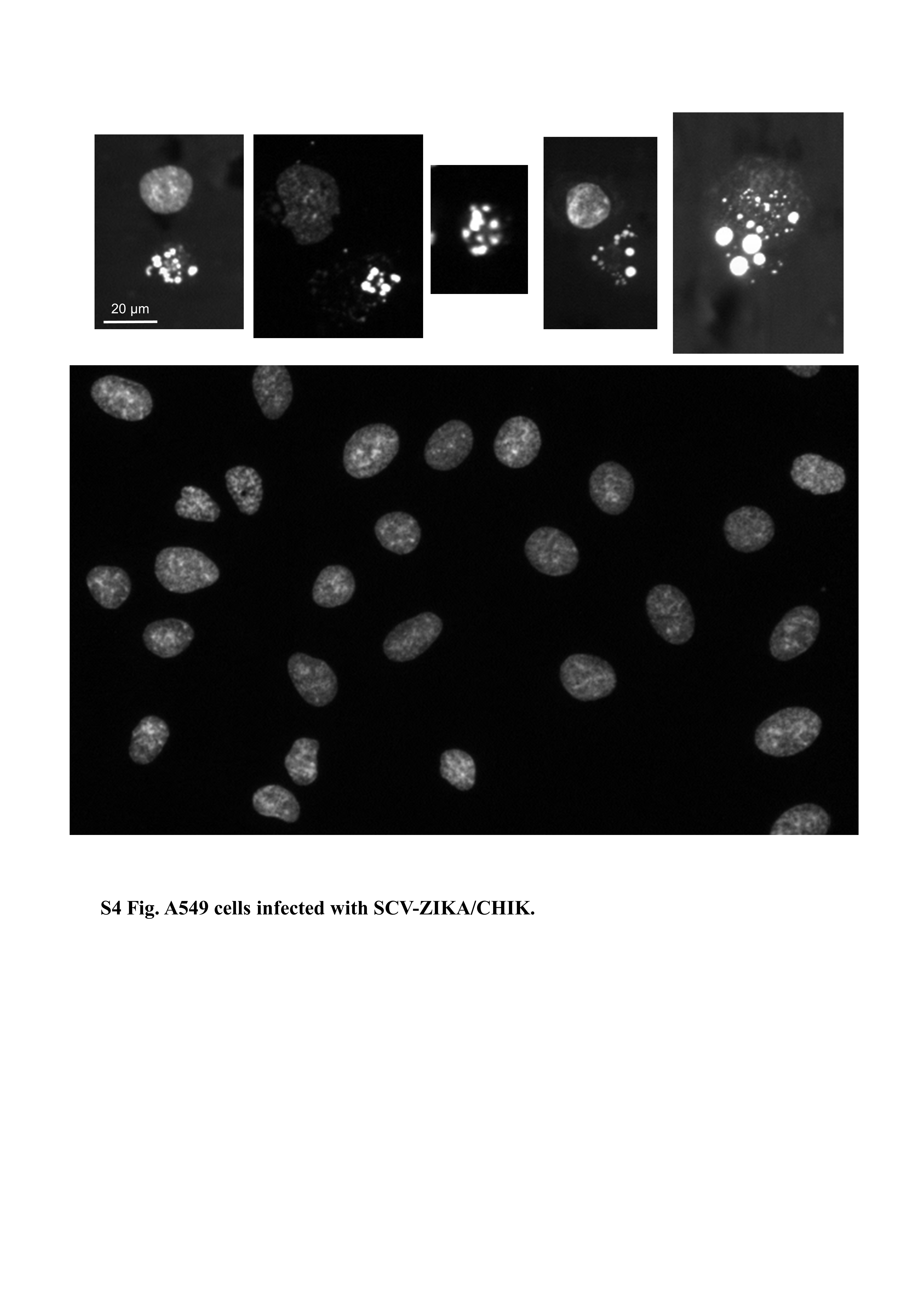

Supplement: S4 Fig — 48–72 hours after SCV-ZIKA/CHIK infection of A549 cells in vitro, morphological features characteristic of apoptosis (condensation of chromatin) were clearly evident (top row) after staining with Hoechst 33342 1. Bottom image shows uninfected controls. 1Linn et al. Complete removal of mycoplasma from viral preparations using solvent extraction. J Virol Methods. 1995. 52(1–2):51–4). (TIF) [file ppat.1009215.s004.TIF]

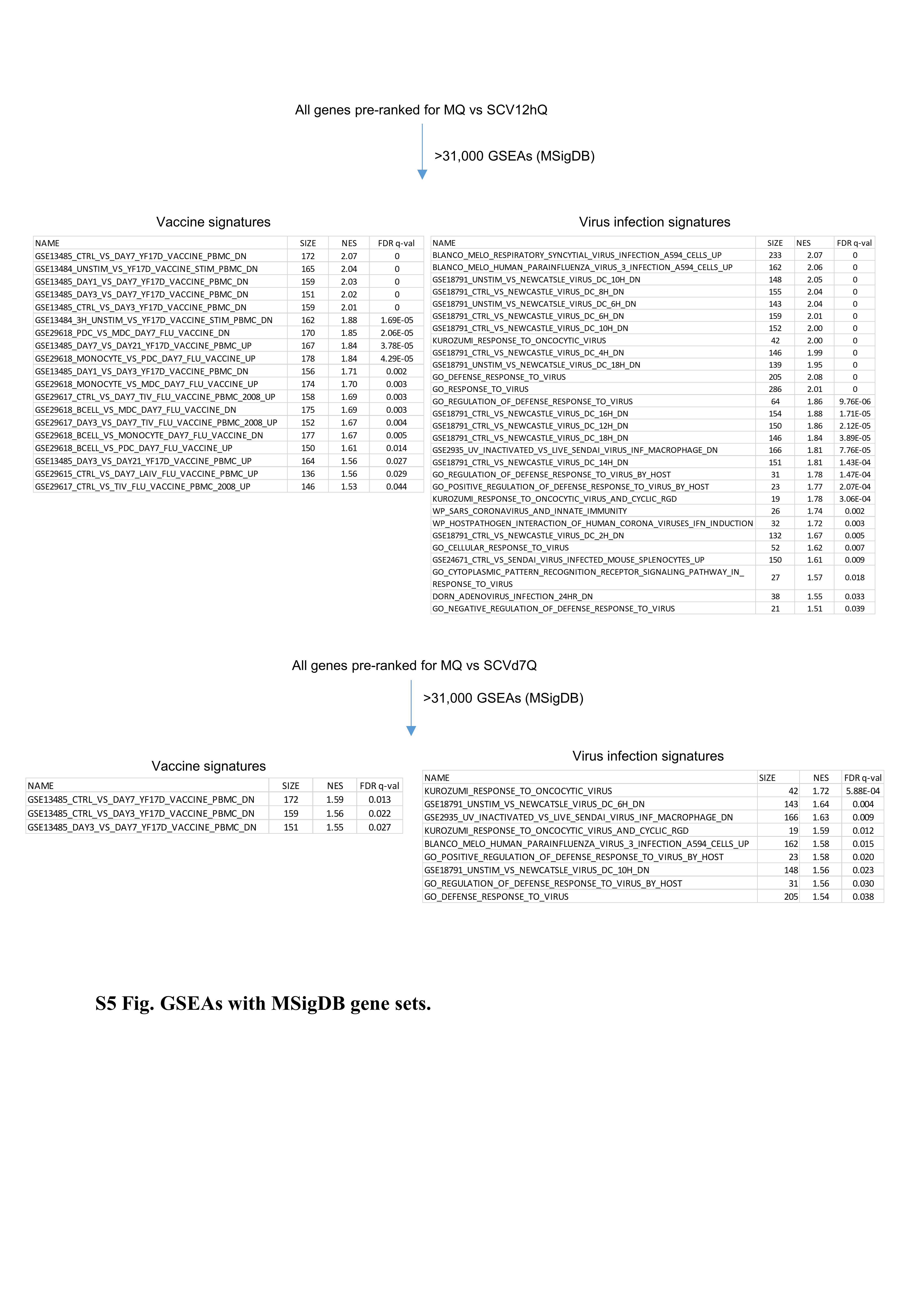

Supplement: S5 Fig — All genes for MQ vs SCV12hQ and MQ vs SCVd7Q were pre-ranked and GSEAs run for the >31,000 genes set available in MSigDB. Listed are vaccine and virus infection signatures where q<0.05. (TIF) [file ppat.1009215.s005.TIF]

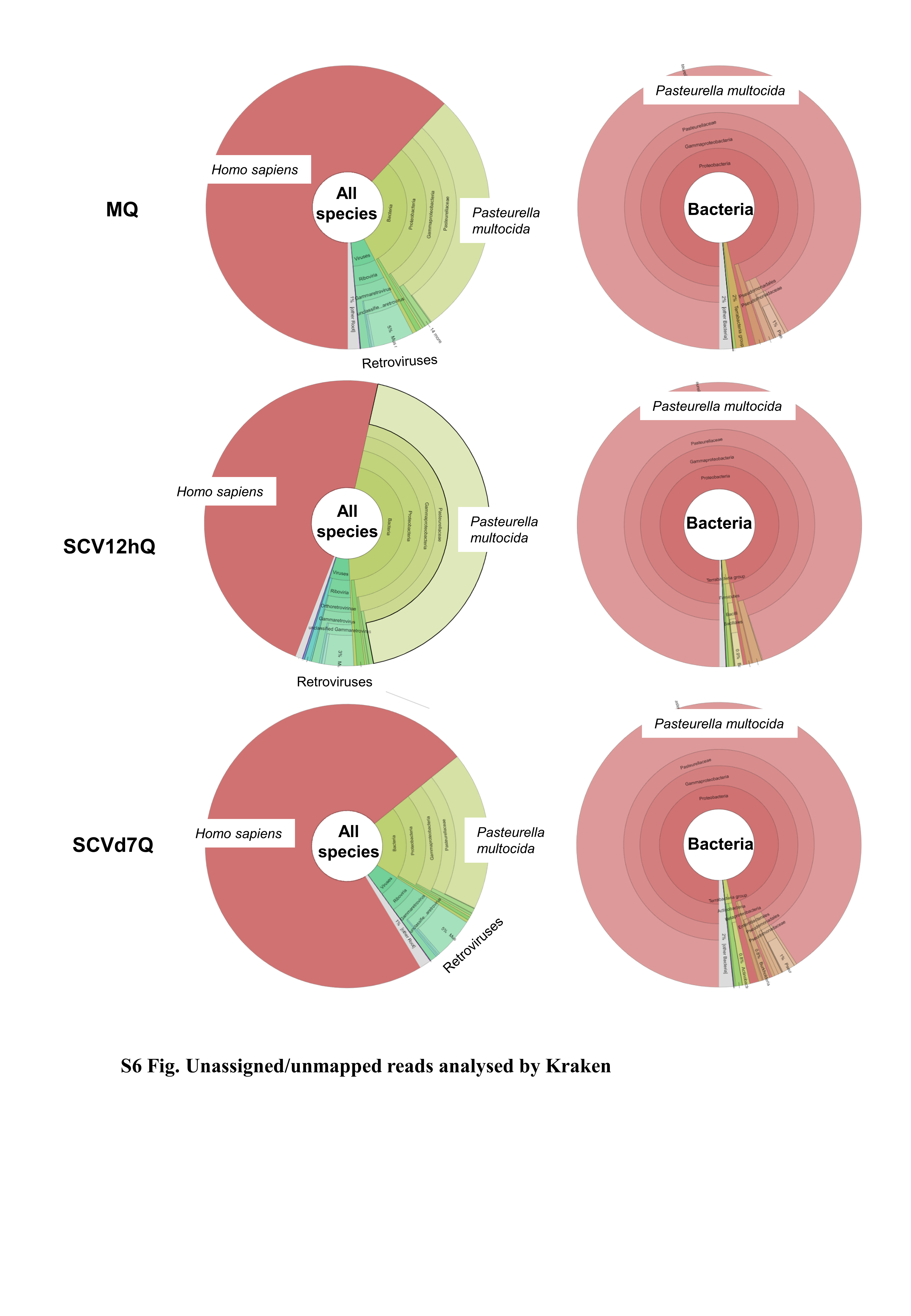

Supplement: S6 Fig — Reads that were not assigned by the STAR aligner to mouse or vaccine genomes for MQ, SCV12hQ and SCVd7Q were analysed by Kraken, a metagenomic sequence classification tool. The output for all classified reads (All) was dominated by human sequences (Homo sapiens), Pasteurella multocida (a commensal of dogs, cats and rabbits), and murine retroviruses (primarily Mus musculus mobilized endogenous polytropic provirus). Mice in our animal house facility routinely test negative for Pasteurella species, so this contamination is unlikely to have originated from the mice. About 10% of the mouse genome is made up of endogenous retroviruses, with multi-mapped reads (reads that align to multiple locations in the mouse genome) left unassigned by the STAR aligner. (TIF) [file ppat.1009215.s006.TIF]
