# Supplementary material for: Injection site vaccinology of a recombinant vaccinia-based vector reveals diverse innate immune signatures
Source: PLoS Pathog. 2021 Jan 13;17(1):e1009215. doi: 10.1371/journal.ppat.1009215 (PMC7837487; doi:10.1371/journal.ppat.1009215)
Supplement: Attachment — Submitted filename: Response_to_reviewers.docx [file ppat.1009215.s009.docx]

**PPATHOGENS-D-20-01887 Detailed response to reviewers’ comments**

“Injection site vaccinology of a recombinant vaccinia-based vector reveals diverse innate immune signatures”.

The reviewers’ comments have been reproduced in their entirely in italics to facilitate navigation of the numerous issues raised. The addition bioinformatics work conducted to address reviewers comments has resulted in an addition author, CR Bishop.

***Part I - Summary*** *Please use this section to discuss strengths/weaknesses of study, novelty/significance, general execution and scholarship.*

***Reviewer #1:*** *In this manuscript the authors have studied the changes in gene expression after vaccination in mice with a multiplication defective poxvirus-based vector system called Sementis Copenhagen vector. This vector expresses the structural genes of both CHIKV and ZIKV as immunogen. The vaccination was done by intramuscular injection (IM) and muscles were harvested at 12 hours and 7 days post vaccination. The authors performed RNA-Seq and bioinformatics to characterize the injection site innate responses and expression of the recombinant immunogens.
This reviewer thinks there are several issues with the study design:*

*1. The authors should add vector only control, to differentiate the changes that are due to the expression of immunogens. It is known that the vector itself will activate or suppress many of the innate response markers.*

We agree this is a potential shortcoming of this study and we have added a paragraph in the discussion to this effect, but have also highlighted the difficulties in separating vector from immunogen signatures. The SCV vector itself differs from SCV-ZIKA/CHIK in a number of ways, not just expression of the immunogens; specifically SCV-ZIKA/CHIK (i) has the immune modulating genes A39R, B7R/B8R deleted, and (ii) has two synthetic strong early late promoters. Although SCV (i.e. Copenhagen strain of VACV with D13L deleted) might be considered as a potential smallpox vaccine candidate, at this stage, we have not manufactured, purified and completed release tests for SCV to the same standard as we have undertaken for SCV-ZIKA/CHIK. The latter process is now briefly explained in a new section in the Methods and was undertaken to meet the standards required for NHP testing at the NIAID (Prow et al 2020). Manufacture of SCV to the same standard represents a considerable undertaking and would be required before one could meaningfully do side by side comparisons between SCV and SCV-ZIKA/CHIK.

To be able to separate the immunogen signatures from SCV signatures for SCV-ZIKA/CHIK one would perhaps optimally need to construct a new vaccine with stop codons introduced into the immunogen coding sequences, and then perform RNA-Seq. Only in this way could the influence of A39R, B7R/B8R, synthetic strong early late promoters, immunogen inserts, VLPs etc be clearly elucidated. However, this again represents a very considerable undertaking.

Given the concordance with existing poxvirus literature (S2E Table) and the known poor immunogenicity of recombinant proteins, the dominant signatures likely arise from the vector rather than the immunogens. We have added a section in the Discussion to elaborate on this contention. To avoid batch effects and see small differences, comparisons between SCV-ZIKA/CHIK, SCV-ZIKA^STOP^/CHIK^STOP^, and SCV would have to be run together, otherwise the role of the immunogens could not be cleanly separated.

We thus contend that such experiments constitute a very large undertaking, would double the amount of data to be presented, and is in our view beyond the scope of the current paper.

*2. Does analysis of muscle only (injection site) represent systems vaccinology?*

We agree with the reviewer that the term “systems vaccinology” is perhaps somewhat confusing as it is unclear what “system” means in this context. We have thus replaced this phrase with “injection site vaccinology” to clearly describe the objective of the study.

*What about detection of immune responses in other key organs? B cells, T cells?*

SCV-ZIKA/CHIK is immunogenic in mice and non-human primates, thus cognate immune responses in spleen and lymph nodes would be entirely expected. Such studies (described as systems vaccinology studies) have now been completed for a large number of settings, but provide minimal information on adjuvant signatures and injection site responses; the two features that were the focus of our study. By removing the words “system vaccinology” (see above), hopefully this focus is now clearer.

A key organ for the SCV-ZIKA/CHIK vaccine is arguably the joints, given the past history of arthralgia for a several MVA and a previous CHIKV vaccine. No compelling signatures were identified in feet (new Fig. 6A), arguing that this vaccine does not cause arthropathy.

*It is not surprising that at the injection site after 7 days almost nothing is detected.*

Injection site reactions are the most common adverse reactions observed in clinical trials of MVA, so this remains an issue for regulators. Thus, although perhaps expected, we needed formally to characterise the behaviour of the vaccine on day 7. We have added the phrase “with acute resolving injection site reactions also the most common adverse reactions observed in clinical trials of MVA {, #153}” to the introduction to clarify the rationale.

It is indeed perhaps not surprising that no vaccine mRNA is detected, given previous studies on MVA, where luciferase expression by MVA is lost 48 hours post inoculation. We have thus added a sentence to the results (**Read alignments to the SCV-ZIKA/CHIK vaccine genome**) to illustrate that our observations are indeed consistent with those reported for MVA. We nevertheless needed to formally provide the evidence for SCV.

*3. What was the rationale of selecting only these two time points?*

This is explained in detail in the last paragraph of the Introduction. We have expanded this to include the arthralgia noted in MVA vaccine trials. We could not readily see any rational basis for including additional time points, given the complexity already inherent in the current analyses.

*4. Validation of data?*

In the main, this paper makes claims regarding pathways, rather than individual genes. Pathway identification relied heavily (in this case) on annotations within IPA/Cytoscape. To provide an independent process, we used the entire gene lists (not just the DEGs) to interrogate the >31,000 gene sets in MSigDB using GSEAs. The results are presented in a new section in the Results (**Concordance with vaccine and virus infection gene sets**) and S5 Fig. Essentially, the output showed highly significant concordance of our data with multiple vaccine and virus-infection signatures. We contend that this analysis using >31,000 GSEAs provides a powerful validation of our RNA-Seq data.

We have also added a new MDS plot in S1E Fig to illustrate the tight clustering of replicates for muscle and the clear segregation between groups (with the exception of MF and SCCd7F where very few DEGs were identified). This constitutes an additional quality control for the RNA-Seq data.

The data presented in Fig 1E and the concordances described in S2E Table might also be viewed as validation of the RNA-Seq data, as they constitute independent evidence for the existence of pathways identified by IPA/Cytoscape.

***Reviewer #2:*** *The authors of this manuscript used RNAseq to analyze changes in the abundance of gene transcripts in mice vaccinated (IM) with a recombinant VAC strain encoding Chikungunya and Zika virus antigens. Two time points were evaluated, 12 hr and 7 days post-vaccination. Although the conclusions were perhaps not too surprising, the investigation and subsequent analyses provided a comprehensive snapshot of the innate and adaptive response to such an infection. I found this an interesting manuscript, excellent science, and intriguing observations. The results would be of interest to anyone trying to understand how a recombinant poxvirus might vector an antigen. Lots to chew on.
My criticisms are not so much scientific as concerning the manuscript itself. As a general observation it often reads like a review of vaccinia virus gene functions, which has value, but at times isn't too relevant to the interpretation of these data.*

*In particular there are many references to how VACV protein "X" inhibits protein "Y", but then it is often unclear why that should have any impact on the level of the relevant cellular transcript(s). For example, on lines 272-3 it's said that "The DNA sensor PRKDC/DNA-PK has a z-score of zero, perhaps due to the inhibitory activity of C4 (encoded by C4L)...". Binding wouldn't necessarily affect transcript levels. Unless it's known that there is a feedback loop wherein the stated virus-host interaction inhibits expression of its binding target, such an interaction isn't really of relevance to the paper.*

The expression of PRKDC/DNA-PK mRNA is indeed not changed (S2D Table), so no feedback needs to be invoked. IPA USR z scores reflect the transcriptional activity of the upstream regulator, which can, but often do not depend on, changes in expression of that regulator. We have added the sentence “The expression of PRKDC/DNA-PK mRNA was not significantly altered (S2D Table), consistent with C4 protein-protein interactions {Scutts, 2018 #150} inhibiting the transcriptional modulation activity mediated by this upstream regulator.” to clarify the bioinformatic interpretation.

*In a related discussion (lines 316-9), the authors note that K1 and A49 block NFkB signaling in the context of the fact that IPA USR analysis detected a "dominant NF-kB signature". Doesn't such a signature imply that such promoter elements are active? This seems to be a non-sequitur, unless one is willing to hypothesize that K1 and A49 aren't actually working as we suppose.*

We have added the following sentence to clarify our interpretation of these analyses; “As the SCV-encoded inhibitors of NF-κB signaling are expressed only in the SCV-infected cells, the dominant NF-κB signatures are likely largely associated with uninfected cells stimulated and/or recruited by the pro-inflammatory environment.” So K1 and A49 may block NF-κB signaling in SCV-infected cells, but clearly cannot block NF-κB signaling in, for instance, the recruited neutrophils. There is thus no *non-sequitur*, or a need to argue that K1 and A49 are inactive; nevertheless, the reviewer is correct in highlighting the need to be clear that this analysis cannot separate what is happening in infected versus uninfected cells.

*I would ask that the authors take a close look at wherever they have described a VACV gene function, and ask themselves whether the citations (over 200 of them) help the reader gain a better understanding of the data reported in this manuscript.*
RNA-Seq by its very nature describes the entire transcriptomic changes and thus a full account of all significant results necessarily ensues. A large body of literature becomes relevant to the observations. In fact reviewers have pointed where we have missed several pertinent references, which we have now added.

*Some minor observations:
line 72-3. Should ACAM2000 be included? It's arguably more relevant than racoonpox.*

We have added ACAM2000 with a reference to the Introduction. We are repeatedly requested to add raccoonpox.

*line 123. Are there bacterial reads in the remaining 8.4%? That is, is there any signature associated with secondary microbial infection?*

This is an excellent point and to address this issue we have added a section in the Results to describe the sterility and mycoplasma testing that the vaccine had undergone prior to vaccination of the mice (and NHPs in another study).

We have added a Kraken metagenomic analysis to the Supplementary Figures to analyse what the unmapped or unassigned reads from the RNA-Seq analyses might be. Not surprisingly we found some contaminations, but importantly there was no indication of injections site infections. This is actually a very useful additional readout for injection site vaccination and we thank the reviewer for suggesting this analysis. A new section in the Results covers this new analysis “**No detection of adventitious agents or microbial infections**“.

*line 154. Should probably read "gene" not "protein"*

This error has been corrected.

*line 177. Unless I missed it, somewhere it should be stated explicitly that all the reported effects were absent in mock vaccinated animals.*

We have added “No significant staining was observed in MQ” to clarify this point.

*line 249-50. The sentence is so full of abbreviations as to render it incomprehensible. There are others like it. Are all of these abbreviations really needed?*

Yes this is perhaps a problem, but we see no way of making this easier.

*lines 526-30. I wasn't quite sure what reason exists to review different adjuvants. Please clarify.*

The underlined section has been added to explain why we deemed this worthwhile “The immunogenicity of SCV, and likely poxvirus vector systems generally, would appear to be underpinned by the ability to stimulate a broad range of pathways known to be stimulated by adjuvants already licensed or being tested in humans”.

This is clearly at the heart of the manuscript and explains why poxvirus systems are so effective as vaccines.

*Fig 5I. Are some of the muscle signatures related to the damage caused by the injection? Is there any data that would allow a comparison of the mock vaccinated (MQ) versus untreated tissue?*

We avoided any mechanical muscle damage signatures by using mock injections so that mechanical muscle damage would be present in both MQ and SCV vaccinated muscles. Such signatures would clearly be unrelated to the vaccine and thus of minimal relevance in an injection site vaccinology study.

***Reviewer #3:*** *Review on Hazlewood et al., PPATHOGENS-D-20-01887
The many observations are extensively discussed and appear sometimes somewhat speculative. Here, it is regrettable that the data analysis just compares the inoculations with SCV-ZIKA/CHIK or saline. From the perspective of poxvirus biology and poxvirus vector vaccine development the study would have been even more informative when including the analysis of inoculations with the original Copenhagen strain of VACV and with an empty SCV. The following specific points should be considered:*

**Part II – Major Issues: Key Experiments Required for Acceptance**

***Reviewer #1:*** *This reviewer thinks there are several issues with the study design:
1. The authors should add vector only control, to differentiate the changes that are due to the expression of immunogens. It is known that the vector itself will activate or suppress many of the innate response markers.*

This comment was raised in Part I – and was addressed above.

*2. Does analysis of muscle only (injection site) represent systems vaccinology?*

We have now referred to this study as injection site vaccinology

*What about detection of immune responses in other key organs? B cells, T cells? It is not surprising that at the injection site after 7 days almost nothing is detected.*

This comment was raised in Part I – and was addressed above.

*3. What was the rationale of selecting only these two time points?*

This comment was raised in Part I – and was addressed above.

*4. Validation of data?*

Addresses previously (please see above)

***Reviewer #3:*** *1. Results (p7, ln 153). The promising finding of abundant recombinant immunogen transcripts (~20%) among the total viral transcripts would deserve further attention. It would be interesting to determine if the particular replication-defective life cycle of SCV contributes to this expression phenotype.*
Although perhaps interesting to determine whether D13L deletion would increase the activity of the synthetic strong early late promoters, we can imagine no reasons why this would occur; especially at 12 hr when virus replication would be minimal in both groups. Certainly not a straightforward experiment to undertake, given the very extensive state and federal safety regulations that have now been imposed on the use of replication competent vaccinia.

*2. Results, histopathology (Fig. 1D). The lesions described here appear quite extensive seen the low dose of virus inoculated into the muscle. Larger magnifications of the photograph should be provided to allow for a clear histopathological picture/analysis of the potentially damaged tissue.*

A high resolution image of Fig. 1D is provided in S3A Fig, with pyknotic nuclei indicated. The high resolution image also clearly indicates the loss of striations in the necrotic muscle cells.

*3. Results, immunohistochemistry (Fig. 1F). This type of analysis is always prone to background staining and requires appropriate negative controls. The authors should demonstrate that there is no background staining in muscle tissues infected with empty SCV.*
The background issues are best addressed by staining a parallel section with a control antibody, otherwise the cells infected by SCV cannot be identified and the non-specific staining potentially mediated by the ZIKA/CHIK immunogens is neglected. We provide such a control as a new S2B Fig.

*4. Results (p20-21), presence of eosinophils and absence of arthritic signature (Fig. 5). Is the assumption that the delivery of CHIKV antigens might result in a potentially harmful inflammatory response or in an autoimmune-like reaction (at day 7 post vac)? The rational to ask these questions needs a clear explanation (other than reference to CHIKV infection in general). Is there any evidence of adverse events caused by these subunit antigens? If so, the comparative analysis of empty SCV inoculations seems mandatory to address these questions.*

The reviewer points out an unintended miscommunication on our part, we have extensively restructured the section for Fig. 6 and added a new Fig. 6A. We entirely concur with the sentiment expressed by the reviewer and have thus added the following to the Results section to make this entirely clear “To the best of our knowledge there is no evidence to suggest that CHIKV antigens migrate to joints and cause arthropathy. Instead, CHIKV arthritis {Prow, 2017 #730}, and viral arthritides generally {Suhrbier, 2009 #735}, are associated with replication of virus in the joints, with SCV-ZIKA/CHIK not detected in feet (Fig. 1A). Nevertheless, after the experience with an attenuated CHIKV vaccine (TSI-GSD-218) that caused arthralgia in 5 of 58 vaccine recipients {Edelman, 2000 #186}, arthropathy caused by CHIKV vaccines remains a standing concern for regulators {Schwameis, 2016 #725”.

*5. Discussion (p22, ln 513 ff). The statement that the observed early innate signatures may be shared amongst different poxviruses is interesting but speculative.*

We have rephrased using ‘suggest’ and ‘may’ to highlight the speculative nature of this statement.

*Here, it would have been very interesting to directly compare data e.g. from inoculations with the VACV Copenhagen as the direct ancestor of SCV.*

Indeed, however, aside from the extensive safety provision now imposed on the use of such WT VACV, widespread dissemination within the animal would likely have a profound effect on transcriptional signitures.

**Part III – Minor Issues: Editorial and Data Presentation Modifications**

***Reviewer #3:*** *1. Author summary (p3, ln 58). Recently, a recombinant MVA vaccine has been licensed by the European Commission as part of Janssen’s two component Ebola vector vaccine using ZABDENO together with MVABEA.*

We have included this information in the Introduction with a reference. Indeed good news for pox vectored vaccines!

*2. Results (p7, ln 146). What is the rational to hypothesize that the SCV vaccine following intramuscular inoculation could disseminate and persist in joint tissues (feet)?*

The regulators at the FDA and the NIAID have repeatedly asked us whether SCV-ZIKA/CHIK causes arthralgia/arthritis. We have added the following sentence to the introduction to explain more clearly the rationale; “Several MVA vaccine trials reported transient acute arthralgia as an adverse event {, #153}{Dunachie, 2006 #728}{Tapia, 2016 #729}, with arthropathy associated with new CHIKV vaccines remaining a standing concern for regulators {Schwameis, 2016 #725} after the experience with a live-attenuated CHIKV vaccine {Edelman, 2000 #186}. “

In the Results section” **No compelling arthritic signatures in feet on day 7 post vaccination”** we have also added

“Arthralgia is a common (>1/100 to <1/10) acute transient adverse event in humans after vaccination with IMVANEX {, #153} or recombinant MVA vaccines {Dunachie, 2006 #728}{Tapia, 2016 #729}”

**“**To the best of our knowledge there is no evidence to suggest that CHIKV antigens migrate to joints and cause arthropathy. Instead, CHIKV arthritis {Prow, 2017 #730}, and viral arthritides generally {Suhrbier, 2009 #735}, are associated with replication of virus in the joints, with SCV-ZIKA/CHIK not detected in feet (Fig. 1A). Nevertheless, after the experience with an attenuated CHIKV vaccine (TSI-GSD-218) that caused arthralgia in 5 of 58 vaccine recipients {Edelman, 2000 #186}, arthropathy caused by CHIKV vaccines remains a standing concern for regulators {Schwameis, 2016 #725”.

*3. Discussion (p23, ln 544ff; p25, ln588ff). The identification of vaccine associated reactogenic (or protective) signatures is an often postulated, potentially attractive goal of systems vaccinology approaches. To contribute to this objective it seems still necessary generate data from appropriate comparative experimental settings. E.g. Mutant MVA with an inactivated IL1-ß receptor elicits improved protective immunity and enhanced CD8 T cell responses (Staib 2005, JGV 86:1997; Cottingham 2008 PLoSOne 3:e1638.). Thus, it could be informative to directly compare the signatures of such a mutant virus to conventional MVA.*
Apologies we missed these references and have rephrased the sentence in the Discussion to read “Reintroducing the IL-1 receptors, SPI-2 (B13R) or B16R {Jackson, 2005 #680}, into the vector may affect CD8 T cell responses {Staib, 2005 #726}{Cottingham, 2008 #727}, but may have minimal effects on antibody responses {Legrand, 2004 #581}.”

Indeed we hope this first paper on RNA-Seq injection site vaccinology will spawn more studies.

*4. Discussion (p25, ln576). MVA does replicate its DNA in (most) non-permissive cells.*

We have removed “(unlike MVA)” from this sentence.

*5. Discussion. Reference 193 (p26, ln610) needs checking.*

Our intention with this reference was perhaps somewhat obscure, so we have added the phrase to clarify; “with excessive type I IFN responses associated with adverse events after administration of the yellow fever vaccine*”.*

*6. Discussion (p27, ln632). Recently, a recombinant MVA vaccine has been licensed by the European Commission as part of Janssen’s two component Ebola vector vaccine using ZABDENO together with MVABEA.*

We have added this with a reference to the Discussion and Introduction. Indeed an important development.
